# Imbalance between endothelial damage and repair capacity in chronic obstructive pulmonary disease

**DOI:** 10.1371/journal.pone.0195724

**Published:** 2018-04-19

**Authors:** Jéssica García-Lucio, Victor I. Peinado, Lluís de Jover, Roberto del Pozo, Isabel Blanco, Cristina Bonjoch, Núria Coll-Bonfill, Tanja Paul, Olga Tura-Ceide, Joan Albert Barberà

**Affiliations:** 1 Department of Pulmonary Medicine, Hospital Clínic-Institut d’Investigacions Biomèdiques August Pi i Sunyer (IDIBAPS), University of Barcelona; Barcelona, Spain; 2 Centro de Investigación Biomédica en Red de Enfermedades Respiratorias (CIBERES); Madrid, Spain; 3 Biostatistics Unit, Department of Public Health, School of Medicine, University of Barcelona; Barcelona, Spain; Istituti Clinici Scientifici Maugeri, ITALY

## Abstract

**Background:**

Circulating endothelial microparticles (EMPs) and progenitor cells (PCs) are biological markers of endothelial function and endogenous repair capacity. The study was aimed to investigate whether COPD patients have an imbalance between EMPs to PCs compared to controls and to evaluate the effect of cigarette smoke on these circulating markers.

**Methods:**

Circulating EMPs and PCs were determined by flow cytometry in 27 nonsmokers, 20 smokers and 61 COPD patients with moderate to severe airflow obstruction. We compared total EMPs (CD31^+^CD42b^-^), apoptotic if they co-expressed Annexin-V^+^ or activated if they co-expressed CD62E^+^, circulating PCs (CD34^+^CD133^+^CD45^+^) and the EMPs/PCs ratio between groups.

**Results:**

COPD patients presented increased levels of total and apoptotic circulating EMPs, and an increased EMPs/PCs ratio, compared with nonsmokers. Women had less circulating PCs than men through all groups and those with COPD showed lower levels of PCs than both control groups. In smokers, circulating EMPs and PCs did not differ from nonsmokers, being the EMPs/PCs ratio in an intermediate position between COPD and nonsmokers.

**Conclusions:**

We conclude that COPD patients present an imbalance between endothelial damage and repair capacity that might explain the frequent concurrence of cardiovascular disorders. Factors related to the disease itself and gender, rather than cigarette smoking, may account for this imbalance.

## Introduction

Chronic obstructive pulmonary disease (COPD) is a life-threatening lung disease with systemic impact [1]. The primary cause of COPD is cigarette smoking, which is known to also produce endothelial dysfunction [2]. Previously, we demonstrated that COPD patients show endothelial impairment in both the pulmonary and the systemic circulation that may predispose to pulmonary hypertension (PH) and/or cardiovascular events [3–5]. Circulating biomarkers have emerged as promising non-invasive surrogates which may provide insights on endothelial function status and reveal mechanisms of endothelial derangement. Cellular biomarkers such as endothelial microparticles and progenitor cells have recently been related to endothelial health [6].

Circulating endothelial microparticles (EMPs) are small membrane-bound vesicles (0.1–1μm diameter) shed from the endothelium in response to cell activation, injury and/or apoptosis [6–8]. As they circulate in the vasculature, they not only act on their local environment but also on sites far from their origin, serving as a cell-to-cell communication network [9]. In plasma of healthy subjects, EMPs are present at low levels, reflecting normal endothelial turnover [10]. Increased levels of EMPs have been reported in a variety of vascular-related disorders [7] and their levels are inversely correlated with endothelial function [11]. Cigarette smoke may alter the levels of circulating EMPs [12–15]. In COPD, elevated EMPs levels have been reported [16,17]. Higher EMPs levels with activated phenotype may denote COPD patients susceptible to an exacerbation episode [18], and to be predictive of rapid FEV_1_ decline [19]. Recently, a negative correlation between EMPs in sputum and FEV_1_ has been described in COPD [20].

Circulating progenitor cells (PCs) are adult stem cells derived from the bone marrow that are mobilized into the circulation in response to vascular injury [21]. They are involved in maintenance of the endothelium and restoration of its normal function [21]. However, it has also been reported that PCs could participate in the progression of pre-existing lesions [22]. In a recent study, we showed that COPD patients have lower levels of circulating PCs which appear to be a consequence of the disease itself and not related to smoking habit [5]. In this line, other studies also showed lower PCs levels in COPD [23–28], while others did not find significant differences [29,30]. Decreasing number of circulating PCs has been established as an independent prognostic risk factor associated with endothelial dysfunction and higher cardiovascular risk [31].

As the magnitude of endothelial damage may result from an imbalance between injury and repair capacity, the combined assessment of circulating EMP and PC levels may be used to evaluate the status of vascular health in different disorders [32–34].

Based on this background, we hypothesized that in COPD, alterations of endothelial function are associated with changes in the number of circulating EMPs and PCs. Accordingly, the present study aims to investigate whether COPD patients have an imbalance between EMPs to PCs compared to nonsmokers and current smokers, and the relationship with COPD severity and the presence of PH.

## Methods

### Study population

Sixty-one patients with COPD and 47 control subjects (27 nonsmokers and 20 current smokers) with normal lung function were enrolled in the study. Patients were recruited from the outpatient clinic and nonsmokers and current smokers were volunteers. The study was approved by the internal review board and all subjects gave written informed consent. Patients with left ventricle dysfunction in echocardiography were excluded. Patients were clinically stable at the time of the study without exacerbation episodes or oral steroid treatment for the previous 4 months. All patients were on regular bronchodilator treatment and most of them received inhaled corticosteroids. In control subjects, the absence of lung disease was confirmed by clinical evaluation and lung function tests. COPD was diagnosed according to current guidelines [1]. All subjects underwent standard evaluation by means of medical history, clinical examination, lung function tests and electrocardiogram. COPD was defined by clinical history compatible and evidence of chronic airflow limitation on spirometry (post-bronchodilator forced expiratory volume in the first second (FEV_1_) / forced vital capacity (FVC) < 70%). COPD patients underwent additional diagnostics for the assessment of associated PH; defined on the basis of right heart catheterization (mean pulmonary arterial pressure ≥25 mmHg and pulmonary artery occlusion pressure ≤15 mmHg) or by Doppler echocardiography (tricuspid regurgitation velocity ≥2.8 m/s).

### Blood sampling and measurements

Venous blood samples were obtained after fasting overnight peripheral venipuncture into two 4.5mL sodium citrate tubes (Becton Dickinson, Plymouth, UK) to measure circulating EMPs, into two 4mL tubes with EDTA (Becton Dickinson, Plymouth, UK) to measure circulating PCs and into two 4mL tubes with EDTA for other biochemical determinations. The latter were centrifuged immediately 10min (800g, 4°C) and, after centrifugation, plasma was aliquoted and stored at -80°C until analysis. Plasma levels of vascular endothelial growth factor (VEGF), interleukin-6 (IL-6), ultra-sensible C-reactive protein (hsCRP), transforming growth factor beta (TGF-β), brain natriuretic peptide (BNP), fibrinogen, hepatocyte growth factor (HGF), letpine, adiponection, cyclic guanosine monophosphate (cGMP), soluble tumor necrosis factor-a receptor type I (sTNFa-RI), soluble intercellular adhesion molecule-1 (sICAM-1) and soluble tyrosine kinase receptor Axl (sAXL) were determined by ELISA using commercially available kits (DuoSet, R&D Systems, Abingdon, UK).

#### Assessment of circulating endothelial microparticles

Circulating EMPs were assessed by flow cytometry by the expression of the platelet endothelium adhesion molecule-1 (PECAM-1) marker CD31 in the absence of the platelet-specific glycoprotein Ib marker CD42b (S1A–S1B Fig) [12]. To further evaluate whether EMPs were derived from apoptotic or activated endothelial cells, EMPs were also assessed by annexin V staining for the presence of phosphatidylserine, a marker linked to apoptosis (S1C Fig) [8,12,35] or assessed by CD62E (E-selectin) staining, a cell adhesion molecule expressed only on endothelial cells activated by cytokines (S1D Fig) [8,36,37]. Briefly, peripheral blood was collected and within 1 hour was centrifuged 10 minutes (800g, 4°C) to prepare platelet rich plasma. Within 5 minutes, the supernatant was subsequently centrifuged 10 minutes (300g, 23°C) to discard cells, 10 minutes (2.000g, 23°C) to discard dead cells and finally 30 minutes (10.000g, 23°C) to discard cell debris and to obtain platelet-poor plasma (PPP). EMP phenotype analysis was performed based on size and fluorescence (S1A Fig). Events less than 1 μm were identified in forward (size) and side (density) light scatter plots using size calibration microspheres (FluoSpheres®carboxylate-modified microspheres 1.0μm, yellow-green fluorescent (505/515), Invitrogen, Oregon, EEUU). MPs levels were assessed by comparison with calibrator beads (Perfect Count Microspheres, Cytognos, Salamanca, Spain) with a known concentration, using 2.000 events beads-PE as a stop time. Then 100.000 MPs/μL for fluorescent minus one (FMO) tubes and 500.000 MPs/μL for each phenotype were stained 45 minutes at room temperature using pre-conjugated anti-human monoclonal antibodies and isotype controls: anti-CD31-FITC (BD Pharmingen^TM^, San Jose, CA), anti-CD42b-PE (BD Pharmingen^TM^, San Jose, CA), anti-CD62E-APC (BD Pharmingen^TM^, San Jose, CA), anti-IgG1 k-PE isotype control and anti-IgG1k-APC isotype control both from (BD Pharmingen^TM^, San Jose, CA) for the activated phenotype. For the apoptotic phenotype, MPs without anti-CD62E-APC were additionally labeled using annexin V-APC (BD Pharmingen^TM^, San Jose, CA) in the presence of CaCl_2_ (25mM) according to manufacturer’s recommendation. The fluorescence minus one technique was employed to provide negative controls [38]. Samples were analyzed by two- or three-color fluorescence histograms as CD31^+^CD42b^-^ (S1B Fig), CD31^+^CD42b^-^AnnexinV^+^ (S1C Fig) or CD31^+^CD42b^-^CD62E^+^ (S1D Fig) microparticles. Single antibody conjugates and compensation fluorochrome beads were used for compensation assessment. Samples were acquired at band pass filters: 530 nm (FITC), 585 nm (PE/PI), and 661 nm (APC) with FL4 option. EMPs were quantified by flow cytometry using LRSFortessa (BD Bioscience, San Jose, CA) and 100.000 MPs/ events were acquired. The data were analyzed using FACSDIVA (Tree Star, OR).

#### Assessment of circulating progenitor cells

The number of circulating progenitor cells was evaluated by flow cytometry using antibodies against CD45 (pan-leukocyte marker), CD133 (sub-population of hematopoietic stem cells) and CD34 (mature and progenitor endothelial cells) as previously described [5]. In brief, circulating progenitor cells were isolated from fresh peripheral blood by Ficoll density gradient centrifugation, washed once with phosphate buffered saline (PBS) supplemented with 2% of fetal calf serum (FCS) and ressuspended at 2x10^6^ cells for FMO tubes and at 4x10^6^ cells for sample tubes. Circulating PCs were stained and analyzed by flow cytometry for phenotypic expression of surface markers using pre-conjugated anti-human monoclonal antibodies and isotype controls anti-CD45-FITC (BD Pharmingen^TM^, San Jose, CA), anti-CD34-PECy7 (eBiosciences, San Diego, CA), anti-CD133-PE (MACS Miltenyi Biotec, Bergisch Gladbach, Germany), anti-IgG1k-PECy7 isotype control (eBiosciences, San Diego, CA), anti-IgG1k-FITC isotype control (BD Pharmingen^TM^, San Jose, CA) and anti-IgG1k-PE isotype control (BD Pharmingen^TM^, San Jose, CA). The fluorescence minus one technique was employed to provide negative controls [38]. After 45 minutes of incubation, cells were washed, ressuspended into 500 μL of PBS + 2%FCS and proceeded to flow cytometry analysis. A total of 750.000 CD45+ events were run through the LRSFortessa (BD Bioscience, San Jose, CA). The data were analyzed using FACSDIVA (Tree Star, OR) following ISHAGE (International Society of Hematotherapy and Graft Engineering) gating strategy (S2 Fig) [39].

### Statistical analysis

In the non-adjusted analysis, data are expressed as mean±SD for normally distributed data or as median with interquartile range for skewed distributions. Group comparisons were performed using one or two way ANOVA and post hoc pairwise comparisons using the Student Newman-Keuls test for normally distributed variables or the Dunn’s test for non-normally distributed variables. Correlations between variables were analyzed with Pearson’s or Spearman’s coefficient depending on data distribution.

In the statistical adjusted model, as EMP and PC counts were skewed in distribution, values were ln-transformed to improve normality. Linear regression models were used to adjust for potential confounders, which were selected based on biologic plausibility such as age, gender, body mass index (BMI) and Framingham risk score [40].

A p-value <0.05 was considered statistically significant.

## Results

### Population characteristics

Anthropometric, clinical and functional characteristics of subjects are shown in Table 1. Nonsmokers and current smokers were matched for age, gender and BMI. COPD patients were older and with a higher proportion of men. All healthy smokers and 26% of COPD patients were current smokers. The COPD group had higher Framingham risk score compared with the other groups. Three patients with COPD (5%) were in spirometric GOLD stage 1, 17 (28%) in stage 2, 18 (29%) in stage 3, and 23 (38%) in stage 4. Patients with COPD had moderate to severe airflow obstruction, air trapping, reduction of CO diffusing capacity and mild to moderate hypoxemia (Table 1). Characteristics of subjects by gender are shown in S1 Table.

**Table 1:** Clinical characteristics, lung function, cardiovascular and laboratory measurements.

	Nonsmokers (n = 27)	Current smokers (n = 20)	COPD patients (n = 61)
Age, years	56 ± 8	54 ± 8	63 ± 7*^#^
Male gender, n(%)	12 (44)	9 (45)	51 (84)*^#^
BMI, Kg/m^2^	27 ± 3	27 ± 5	27 ± 4
Current smokers, n(%)	0 (0)	20 (100)*	16 (26)*^#^
Former smokers, n(%)	0 (0)	0 (0)	45 (74)*^#^
Smoking history, pack-years	0	30 ± 24*	64 ± 28*^#^
Framingham risk score ‡	5 ± 5	7 ± 6	11 ± 6*^#^
Spirometric GOLD stage 1/2/3/4, n(%)	n/a	n/a	3(5)/17(28)/18(29)/23(38)
FEV_1,_ % predicted	107 ± 12	103 ± 10	44 ± 19*^#^
FVC_,_ % predicted	106 ± 11	104 ± 12	79 ± 19*^#^
FEV_1_/FVC	0.78 ± 0.05	0.77 ± 0.05	0.41 ± 0.14*^#^
TLC, % predicted	105 ± 8	106 ± 9	115 ± 20*^#^
RV, % predicted	108 ± 18	110 ± 23	184 ± 58*^#^
DLco_,_ % predicted	92 ± 15	85 ± 9	53 ± 20 *^#^
PaO_2,_ mmHg	n/a	n/a	70 ± 11
PaCO_2_, mmHg	n/a	n/a	44 ± 8
Systolic blood pressure, mmHg	124 ± 18	123 ± 16	131 ± 20
Diastolic blood pressure, mmHg	75 ± 9	73 ± 8	76 ± 11
Total Cholesterol, mg/dL	205 ± 26	218 ± 39	195 ± 34^#^
HDL, mg/dL	57 ± 14	68 ± 15*	58 ± 18
LDL, mg/dL	129 ± 22	132 ± 32	113 ± 30*^#^
Mellitus diabetes, n (%)	1 (4)	0 (0)	7 (11) *^#^
Lymphomonocytes, x10^5^events	8.8 ± 1.4	9.2 ± 2.5	8.4 ± 2.5

Data are shown as mean± SD.

COPD: chronic obstructive pulmonary disease; BMI: body mass index, GOLD: global initiative for chronic obstructive lung disease, FEV_1_: forced expiratory volume in the first second; FVC: forced vital capacity; TLC: total lung capacity; RV: residual volume; DLco: diffusing capacity of the lung for carbon monoxide; PaO_2_: arterial partial oxygen pressure; PaCO_2_: arterial partial carbon dioxide pressure; HDL: high-density lipoprotein; LDL: low-density lipoprotein and NA: not applicable.

‡The Framingham risk score can range from -6 to 19, with higher scores indicating greater cardiovascular risk.

* p< 0.05 compared with control nonsmokers;

^#^ p< 0.05 compared with current smokers.

### Circulating EMPs levels

**Fig 1:**
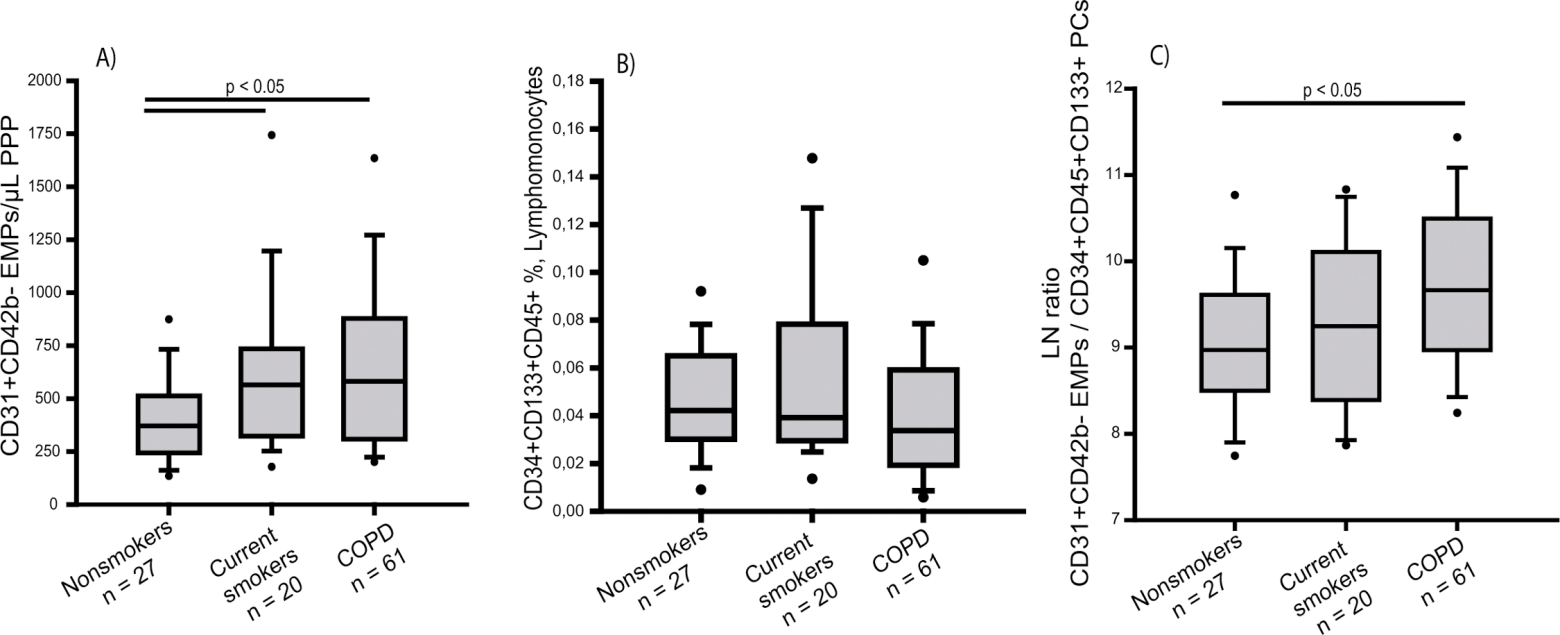
Number of circulating endothelial microparticles (EMPs), progenitor cells (PCs) and the ratio EMPs/PCs in nonsmokers, current smokers, COPD patients. Number of (A) CD31^+^CD42b^-^ EMPs expressed per μL of poor platelet plasma (PPP), (B) CD34^+^CD133^+^CD45^+^ labelled cells expressed as percent of lymphomonocytes and (C) CD31^+^CD42b^-^ EMPs/ CD34^+^CD133^+^CD45^+^ PCs expressed as the ln. The whiskers extend from the box to the 90^th^ and 10^th^ percentiles. Kruskal-Wallis One Way Analysis of Variance on Ranks.

In the non-adjusted model, the number of total circulating EMPs was significantly increased in COPD patients and current smokers compared with nonsmokers (Table 2 and Fig 1A). In the adjusted model, where levels of EMPs were corrected by age, gender, BMI and Framingham risk score, COPD patients also showed significantly increased levels of total circulating EMPs compared with nonsmokers. However, while in current smokers, adjusted levels of EMPs were also higher than in nonsmokers, differences did not reach statistical significance (Table 2). Similarly, levels of EMPs derived from apoptotic origin were increased in both COPD patients and smokers compared with nonsmokers in the non-adjusted analysis (Table 2 and S3A Fig). In the adjusted model, only patients with COPD showed significantly higher number of these EMPs compared to nonsmokers. No statistical differences were found in the levels of EMPs derived from activated endothelial cells between groups, either using the non-adjusted or the adjusted models (Table 2 and S3B Fig).

**Table 2:** Circulating endothelial microparticles counts.

	Nonsmokers (n = 27)	Current smokers (n = 20)	COPD (n = 61)
**CD31+CD42b- EMPs/μL PPP**			
Non-adjusted model (mean, (95% CI))	410 (330–490)	622 (442–801)*	668 (552–785)*
Adjusted model† (predicted mean, (95% CI))	357 (284–448)	526 (404–686)	549 (471–639)*
**CD31+CD42b-Annexin V+ EMPs/μL PPP**			
Non-adjusted model (mean, (95% CI))	290 (230–351)	448 (311–584)*	535 (430–640)*
Adjusted model† (predicted mean, (95% CI))	242 (186–317)	365 (268–498)	414 (346–494)*
**CD31+CD42b-CD62E+ EMPs/μL PPP**			
Non-adjusted model (mean, (95% CI))	14 (6–21)	17 (8–26)	9 (5–14)
Adjusted model† (predicted mean, (95% CI))	5 (3–8)	7 (4–13)	5 (3–7)

* p<0.05 compared to nonsmokers

† Model adjusted for age, gender, BMI and Framingham risk score. Values are expressed as the anti-ln. EMPs: endothelial microparticles, COPD: chronic obstructive pulmonary disease, PPP: platelet poor plasma, BMI: body mass index and CI: confidence interval.

### Circulating PCs levels

Initial analysis of circulating levels of CD34^+^CD133^+^CD45^+^ PCs showed no significant differences between the different groups (Fig 1B). However, analysis of covariates revealed that gender had a significant effect on the levels of circulating PCs. Accordingly; the subsequent assessment of circulating PC levels was performed dividing all groups by gender.

Among women, those with COPD showed significantly lower levels of circulating PCs than both nonsmokers and smokers in the non-adjusted analysis (Table 3 and S3C Fig). Similar findings were observed using CD34+CD133+ and CD34+CD45+ combinations of PCs (data not shown). In men, no significant differences were found in the number of PCs between groups (Table 3 and S3D Fig). In the adjusted model, levels of circulating PCs were reduced in COPD compared to smokers in both men and women (Table 3). No statistical differences were found between the nonsmoker and smoker groups in the number of circulating PCs (Table 3). Interestingly, women showed reduced levels of PCs compared to men throughout all groups, primarily in COPD subjects (p = 0.031) (Table 3).

**Table 3:** Circulating progenitor cell counts.

CD34^+^CD133^+^CD45^+^ (% lymphomonocytes)	Gender	Non-adjusted model (Mean, (95% CI))	Adjusted model† (Predicted mean, (95% CI))
Non-smokers (n = 27)	Men (n = 12)	0.050 (0.035–0.067)	0.062 (0.045–0.085)
	Women (n = 15)	0.044 (0.032–0.056)	0.038 (0.020–0.050)
Current smokers (n = 20)	Men (n = 9)	0.062 (0.028–0.097)	0.076 (0.053–0.106)
	Women (n = 11)	0.053 (0.029–0.078)	0.047 (0.034–0.063)
COPD (n = 61)	Men (n = 51)	0.048 (0.039–0.057)	0.017 (0.011–0.023)*^#^^‡^
	Women (n = 10)	0.045 (0.038–0.053)^§^	0.028 (0.020–0.038)^#^

† Model adjusted for age, gender, BMI and Framingham risk score. Values are expressed as the anti-ln.

* p<0.05 compared with women nonsmokers;

^§^ p<0.05 compared with men smokers;

^#^ p<0.05 compared with women smokers;

^‡^ p<0.05 compared with men COPD.

COPD: chronic obstructive pulmonary disease, CI: confidence interval and BMI: body mass index.

### Assessment of EMPs/PCs ratio

We assessed the ratio of EMPs to PCs as a measure of the balance between endothelial damage and repair capacity. In our series, the EMPs/PCs ratio was increased in COPD patients compared to nonsmokers; being the smokers in an intermediate position that did not significantly differ neither from the nonsmoker nor from the COPD groups (Fig 1C). Further analysis considering the gender, revealed that women with COPD had greater EMPs/PCs ratio compared to control groups (S3E Fig), while no significant differences were found in men (S3F Fig).

### Effect of cigarette smoking on circulating EMP and PC levels

**Fig 2:**
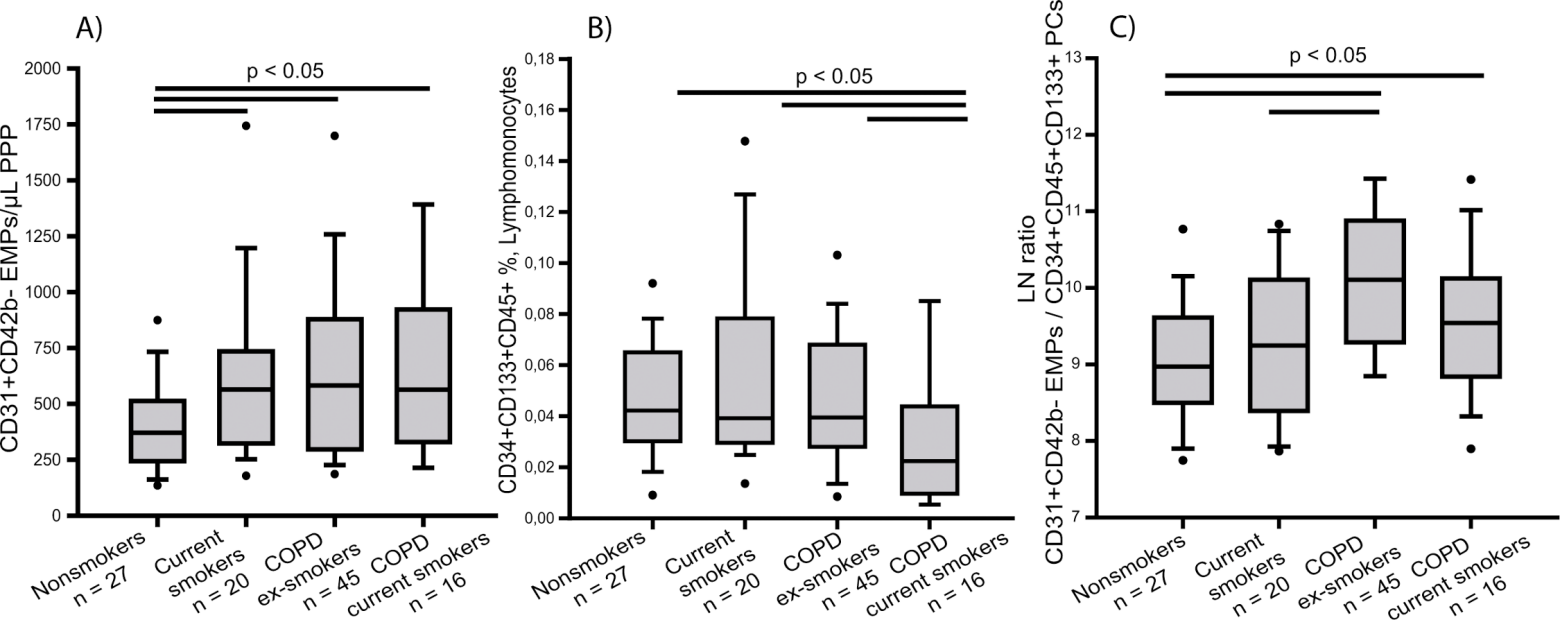
Number of circulating endothelial microparticles (EMPs), progenitor cells (PCs) and the ratio EMPs/PCs in nonsmokers, current smokers, ex-smokers with COPD and current smokers with COPD. Number of (A) CD31^+^CD42b^-^ EMPs expressed per μL of poor platelet plasma (PPP), (B) CD34^+^CD133^+^CD45^+^ labelled cells expressed as percent of lymphomonocytes and (C) CD31^+^CD42b^-^ EMPs/ CD34^+^CD133^+^CD45^+^ PCs expressed as the ln. The whiskers extend from the box to the 90^th^ and 10^th^ percentiles. Kruskal-Wallis One Way Analysis of Variance on Ranks.

Within the COPD group there were both current and ex-smokers. To assess whether smoking status could influence the levels of circulating EMPs and PCs, group comparisons were re-analysed according to smoking status. Our results show that levels of total and apoptotic circulating EMPs were increased in COPD compared to nonsmokers irrespective of smoking status (Fig 2A and S4A).

Regarding circulating PCs, COPD patients that were current smokers had less circulating PCs than the other groups (Fig 2B). When we divided subjects by gender, women with COPD showed a significant reduction of PC levels compared with the other groups, irrespective of the smoking status (S4B Fig), whereas no differences were found in men (S4C Fig).

The EMPs/PCs ratio was higher in COPD patients compared to nonsmokers irrespective of the smoking status (Fig 2C). Similar results were obtained in women (S4D Fig), while in men only COPD patients that were current smokers showed higher EMPs/PCs ratio than nonsmokers (S4E Fig).

### Inflammatory and vascular markers

Compared with nonsmokers and smokers, COPD patients had higher plasma levels of hsPCR, fibrinogen, HGF and siCAM. In the COPD group, VEGF was lower than in the nonsmokers and sTNFα higher than in the smokers (Table 4). No relationship was found between plasma levels of the different biomarkers and the severity of airflow obstruction.

**Table 4:** Vascular and systemic inflammatory markers in the study population.

	Nonsmokers (n = 27)	Current smokers (n = 20)	COPD (n = 61)
VEGF, % detectable	44	60	47
VEGF, pg/mL	47 (23–85)	13 (10–33)	17 (5–32)*
IL-6, pg/mL	1.7 (0,9–5.2)	2.5 (0.9–8.0)	1.7 (0.8–3.1)
hsPCR, mg/dL	0.15 (0,05–0.24)	0.09 (0.05–0.32)	0.38 (0.13–0.86) *^#^
TGF-β, ng/ml	2.3 (1,4–3.4)	2.1 (1.5–5.6)	2.8 2.0–3.6)
BNP, pg/mL	13.7 (4,2–23.4)	12 (8.2–27.7)	15.5 (7.9–27.3)
Fibrinogen, g/L	3.4 (3,1–3.7)	3.3 (2.9–3.8)	3.9 (3.3–4.8) *^#^
HGF, pg/ml	274 219–450)	257 (218–365)	338 (308–418)* ^#^
Leptine, ng/ml	11 (8.5–17.1)	13.9 (6.6–17.4)	11.9 (8.5–21.3)
Adiponectin, ng/ml	1046 (786–1517)	1118 (859–1628)	905 (689–1202)
cGMP, nmol/ml	2.5 (1.9–3.2)	2.2 (1.8–3.0)	2.3 (1.8–321)
sTNFα, pg/ml	1039 (705–411)	897 (691–1159)	1185 (860–1441) ^#^
sICAM, ng/ml	77 (59–95)	86 (67–145)	96 (81–203)*
sAXL, ng/ml	38 (15–71)	42 21–69)	37 (21–60)

Data are shown as median (interquartile range).

VEGF: vascular endothelial growth factor; IL-6: interleukin-6; hsCRP: high sensitive C-reactive protein; TGF-β: transforming growth factor-β; BNP: brain natriuretic peptide; HGF: hepatocyte growth factor, cGMP: cyclic guanosine monophosphate, sTNFα: soluble tumor necrosis factor α; sICAM: soluble intracellular adhesion molecule-1 and sAXL: soluble AXL.

* p < 0.05 compared to nonsmokers;

^#^ p < 0.05 compared to smokers

### Relationship among circulating EMPs and PCs *vs* lung function and cardiovascular parameters

There was no significant correlation between the number of circulating EMPs and PCs, in both the whole population or when divided by gender (S5A–S5C Fig). Circulating EMPs, PCs and the EMPs/PCs ratio were not related to the levels of inflammatory or vascular biomarkers. In the COPD group, no differences in EMP and PC levels or the ratio were observed between those with and without PH.

**Fig 3:**
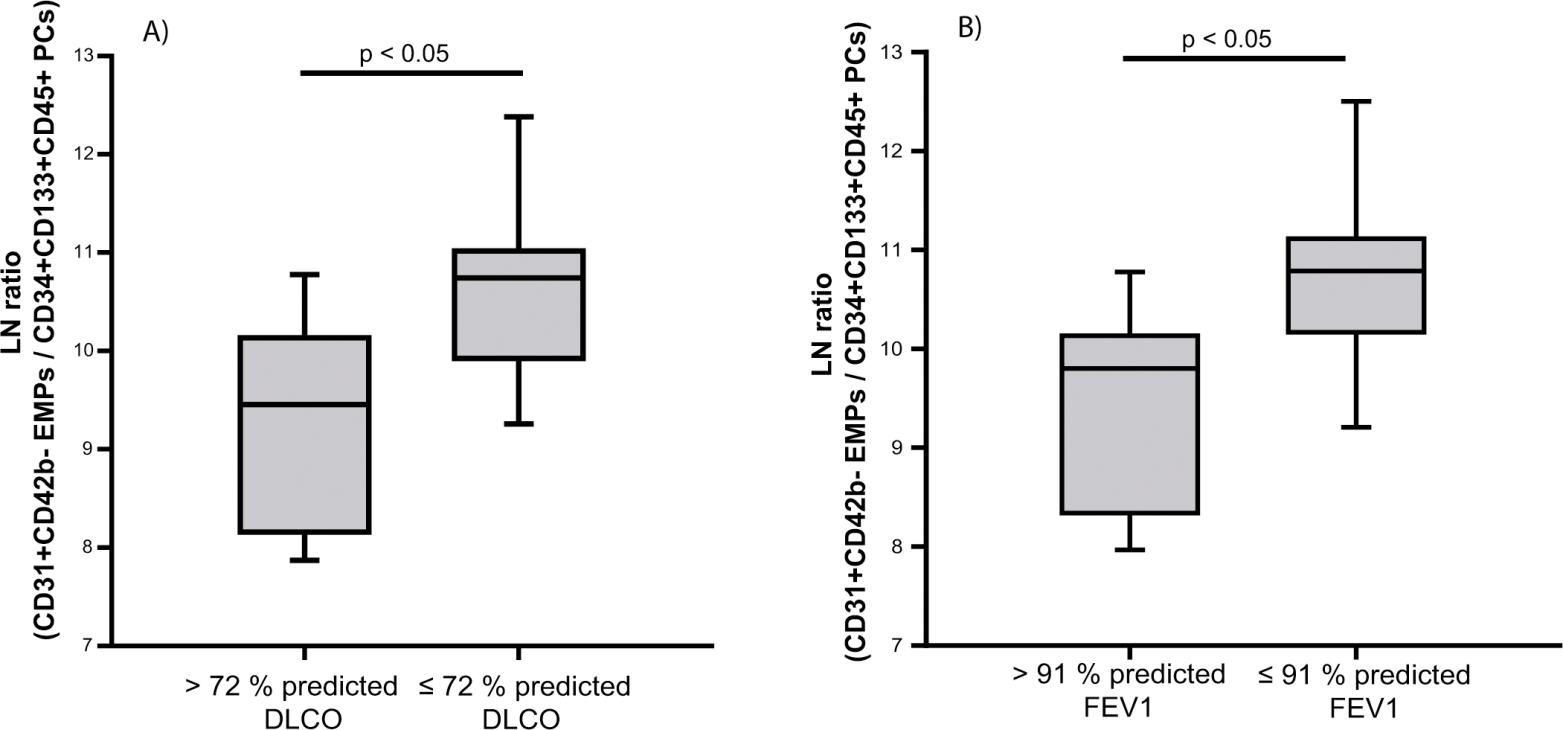
Relationship between the EMPs/PCs ratio and DLco and FEV_1_ in women. Subjects were grouped according to (A) DLco and (B) FEV_1_ above or below the corresponding median value. The box represents the interquartile range and the solid line indicates the median. The whiskers extend from the box to the 90^th^ and 10^th^ percentiles. Ratio values are expressed as ln. Mann-Whitney Rank Sum Test.

In women, the EMPs/PCs ratio was higher in those with CO diffusing capacity (DLco) (Fig 3A), and those with forced expiratory volume in the first second (FEV_1_) below the median (Fig 3B).

## Discussion

In the present study we assessed comprehensively the balance between endothelium injury and repair capacity, which is critical for the maintenance of vascular homeostasis, in patients with COPD. Our results show elevated levels of circulating EMPs, an indicator of endothelial cell damage, and reduced numbers of bone marrow-derived PCs able to maintain and repair the endothelium. The ratio between these two factors was significantly disturbed in COPD suggesting a phenotype of vascular incompetence in those patients [6,33]. Interestingly, while cigarette smoking was not related to this vascular incompetence, gender appears to play a key role on endothelial repair capacity.

In our study the levels of total and apoptotic circulating EMPs were elevated in COPD patients, in agreement with previous observations [16–18]. Since COPD patients showed increased cardiovascular risk factors compared with the other groups, particularly systemic hypertension, and increased cardiovascular risk is associated with greater numbers of circulating EMPs [36], we analysed EMP levels by using an adjusted statistical model to compensate for potential confounding factors. EMP levels in COPD remained significantly higher in the adjusted model, suggesting that increased EMP levels are related to the disease itself rather than to other factors of cardiovascular risk. Interestingly, smokers without COPD also showed significantly higher levels of circulating EMPs than nonsmokers in the non-adjusted analysis but not in the adjusted model. Accordingly, differences in circulating EMPs between smokers and nonsmokers appear to be related to other influencers, i.e. cardiovascular risk factors, rather than to the smoking habit.

In our study, the number of circulating EMPs was not related to indices of COPD severity, suggesting that endothelial damage might be associated with the presence of COPD rather than to its progression. Our results concur with those of Thomashow et al [16] who showed increased numbers of apoptotic EMPs in COPD patients that did not differ among a wide range of COPD severity. Circulating EMPs may act as signalling elements capable of producing endothelial damage in systemic vessels and explain, at least in part, the frequent association between COPD and cardiovascular disease.

Bone marrow-derived circulating PCs are key to endothelial repair [21]. In a recent study, we showed that COPD patients present reduced numbers of circulating PCs [5]. In the present study, COPD patients showed numerically lower numbers of circulating PCs, but differences did not reach statistical significance. Unlike some previous studies in which there was no matching for age and/or gender between groups [24,28], we statistically corrected the data for confounder parameters. Our results revealed that gender had an effect on the levels of circulating PCs in all groups, highlighting the importance of gender matching in PCs studies. In the adjusted model, levels of circulating PCs were reduced in both men and women with COPD. Interestingly, women showed reduced levels of PCs compared to men throughout all groups, primarily in COPD patients. Recent evidence suggests gender-related differences in COPD patients [41]. Women develop more severe COPD at younger ages than men and with lower levels of cigarette smoke exposure [41]. Accordingly, it has been suggested that men and women may be phenotypically different in their response to cigarette smoke [42]. Progenitor cells are actively involved in cardiovascular homeostasis and provide in basal conditions a pool of circulating cells able to repair ongoing endothelial damage. We hypothesize that reduced levels of circulating PCs in women, as compared to men, may result in lower repair capacity and higher susceptibility to the effects of cigarette smoke. In recent years the concept of impaired endothelial cell survival has emerged as a relevant factor in the pathogenesis of emphysema [43]. Although we did not assess the severity of emphysema, in our study a higher EMPs/PCs ratio was associated with lower DLco. Such impairment in the repair capacity of vascular endothelium might explain the more rapid progression of emphysema observed in women [44]. Levels of PCs fluctuate throughout lifetime in women and decline dramatically after menopause, coupled to hormonal mechanisms and endometrial vascular turnover [45]. It has been shown that premature menopause, due to natural or surgical causes, is associated with increased cardiovascular risk compared to non-premature menopause (around 50 years old), suggesting the presence of an estrogen protective effect accumulated during the women´s lifetime [46]. This protective effect on the cardiovascular system is thought to be carried out through the regulation of the endothelial function by the release of nitric oxide and its vasodilation effects [47] All women involved in this study were above 45 years old which could further explain the decline in circulating PCs. Other factors such as genetic predisposition, anatomic, social or hormonal differences might also explain the influence of gender in COPD development [42].

In our study, we did not find any relationship between the levels of circulating EMPs and PCs, suggesting that both markers may reflect different but complementary physiologic cellular mechanisms of action. However, both markers measured together may characterize a phenotype of vascular competence [6,33]. Our study assesses for the first time such vascular competence in COPD patients by the combined measurement of markers of vascular integrity and repair capacity in the same subjects. Our results show a phenotype of disturbed vascular competence in COPD patients, being smokers without COPD in an intermediate position between COPD and nonsmokers. Our findings in COPD are in line with those reported in hypercholesterolemia, psoriasis or Sjögren syndrome [32–34], suggesting a common background for the frequent development of cardiovascular disease in these different conditions. In our study, COPD patients with concomitant pulmonary hypertension did not show differences in EMPs or PCs count, or in the EMPs/PCs ratio, suggesting that such vascular incompetence is insufficient to produce pulmonary hypertension and that additional factors may concur for its development.

Some studies have reported that cigarette smoke increases the levels of circulating MPs [12–15], suggesting early development of emphysema in healthy smokers. The effect of cigarette smoke on PCs is controversial [30,48]. We and others have reported similar numbers of circulating PCs in current smokers and nonsmokers [5,25]. In the present study, there were no differences between total and apoptotic circulating EMPs between former or current smokers. Since differences shown in the present study in COPD patients in circulating levels of EMPs and PCs and in the EMPs/PCs ratio were independent of the current smoking status, we consider that the impairment of vascular competence in COPD appears to be a consequence of the disease itself rather than to an effect of cigarette smoking.

In our study we did not find any relationship between circulating EMPs and PCs and any of the plasmatic markers assessed. This is consistent with recent studies [24] and denotes the complex interactions between markers of systemic inflammation and vascular impairment. In the same line, most of the correlations of EMPs and PCs levels with conventional pulmonary function or demographic parameters were weak or absent.

The study has some limitations. Firstly, as we sampled EMPs in peripheral venous blood we cannot be certain that the elevation of EMPs is from pulmonary origin. Secondly, we did not perform CT scans to assess pulmonary emphysema, therefore we were unable to relate the presence of circulating apoptotic EMPs with emphysematous destruction of lung parenchyma. And thirdly, there is no worldwide exclusive procedure to isolate and analyse circulating MPs and PCs from plasma.

## Conclusions

COPD patients present disturbed vascular competence, as reflected by an imbalance between increased endothelial damage and reduced repair capacity, which might explain the frequent concurrence of cardiovascular disorders. Factors related to the disease itself and gender, rather than the smoking habit, may account for this imbalance.

## Supporting information

S1 Fig Gating strategy for endothelial microparticles (EMPs). A) MPs analysis based on size and fluorescence; B) Sample analyzed by two-color fluorescence histograms as CD31+CD42b- (total EMPs); C) Sample analyzed by three-color fluorescence histograms as CD31+CD42b-Annexin V+ (apoptotic EMPs) and D) Sample analyzed by three-color fluorescence histograms as CD31+CD42b-CD62E+ (activated EMPs).
(EPS)

S2 Fig Gating strategy for progenitor cells (PCs). A) Peripheral blood mononuclear cells (PBMC) selection based on forward and side; B) Singlet selection with no aggregates; C) Sample analyzed by two-color fluorescence histograms as CD34+CD45+ cells and D) Sample analyzed by three-color fluorescence histograms as CD34+CD45+CD133+ cells.
(EPS)

S3 Fig Number of circulating endothelial microparticles (EMPs), progenitor cells (PCs) and the ratio EMPs/PCs in nonsmokers, current smokers and COPD patients. Number of (A) CD31^+^CD42b^-^Annexin V^+^ and (B) CD31^+^CD42b^-^CD62E^+^ EMPs expressed per μL of poor platelet plasma (PPP). Number of CD34^+^CD133^+^CD45^+^ labelled cells expressed as percent of lymphomonocytes (C) in women and (D) in men. Number of CD31^+^CD42b^-^ EMPs/ CD34^+^CD133^+^CD45^+^ PCs expressed as the ln (E) in women and (F) in men. The whiskers extend from the box to the 90^th^ and 10^th^ percentiles. Kruskal-Wallis One Way Analysis of Variance on Ranks.
(EPS)

S4 Fig Number of circulating endothelial microparticles (EMPs), progenitor cells (PCs) and the ratio EMPs/PCs in nonsmokers, current smokers, ex-smokers with COPD and current smokers with COPD. Number of (A) CD31^+^CD42b^-^Annexin V^+^ and (B) CD31^+^CD42b^-^CD62E^+^ EMPs expressed per μL of poor platelet plasma (PPP). Number of CD34^+^CD133^+^CD45^+^ labelled cells expressed as percent of lymphomonocytes (C) in women and (D) in men. Number of CD31^+^CD42b^-^ EMPs/ CD34^+^CD133^+^CD45^+^ PCs expressed as the ln (E) in women and (F) in men. The whiskers extend from the box to the 90^th^ and 10^th^ percentiles. Kruskal-Wallis One Way Analysis of Variance on Ranks.
(EPS)

S5 Fig Correlation between circulating endothelial microparticles (EMPs) and progenitor cells (PCs). Correlation between EMPs and PCs (A) in the whole population, (B) in women and (C) in men. Spearman Rank Order Correlation test. White squares represent nonsmokers, grey triangles represent smokers and black dots represent COPD patients.
(EPS)

S1 Table Clinical characteristics, lung function, cardiovascular and laboratory measurements by gender. Data are shown as mean± SD. COPD: chronic obstructive pulmonary disease; BMI: body mass index, GOLD: global initiative for chronic obstructive lung disease, FEV_1_: forced expiratory volume in the first second; FVC: forced vital capacity; TLC: total lung capacity; RV: residual volume; DLco: diffusing capacity of the lung for carbon monoxide; PaO_2_: arterial partial oxygen pressure; PaCO_2_: arterial partial carbon dioxide pressure; HDL: high-density lipoprotein; LDL: low-density lipoprotein and NA: not applicable. ‡The Framingham risk score can range from -6 to 19, with higher scores indicating greater cardiovascular risk. * p<0.05 compared with men nonsmokers. ^§^ p<0.05 compared with men smokers. ^#^ p<0.05 compared with women nonsmokers. ^¥^ p<0.05 compared with women smokers. ^⁰^ p<0.05 compared with men COPD.
(DOC)
