# Supplementary material for: Imbalance between endothelial damage and repair capacity in chronic obstructive pulmonary disease
Source: PLoS One. 2018 Apr 19;13(4):e0195724. doi: 10.1371/journal.pone.0195724 (PMC5908268; doi:10.1371/journal.pone.0195724)
Supplement: S1 Table — Data are shown as mean± SD. COPD: chronic obstructive pulmonary disease; BMI: body mass index, GOLD: global initiative for chronic obstructive lung disease, FEV1: forced expiratory volume in the first second; FVC: forced vital capacity; TLC: total lung capacity; RV: residual volume; DLco: diffusing capacity of the lung for carbon monoxide; PaO2: arterial partial oxygen pressure; PaCO2: arterial partial carbon dioxide pressure; HDL: high-density lipoprotein; LDL: low-density lipoprotein and NA: not applicable. ‡The Framingham risk score can range from -6 to 19, with higher scores indicating greater cardiovascular risk. * p<0.05 compared with men nonsmokers. § p<0.05 compared with men smokers. # p<0.05 compared with women nonsmokers. ¥ p<0.05 compared with women smokers. ⁰ p<0.05 compared with men COPD. (DOC) [file pone.0195724.s006.doc]

**SUPPORTING INFORMATION**

**RESULTS**

**Population characteristics**

Anthropometric, clinical and functional characteristics of subjects divided by sex are shown in Table S1. Nonsmokers and current smokers were matched for age and BMI in both sexes, however both subsets of COPD patients were older than the respectives control groups. All healthy smokers and 23% of men with COPD and 40% of women with COPD were current smokers. Men had higher Framingham risk score compared to women throughout all groups. (Table 1S).

**Table S1. Clinical characteristics, lung function, cardiovascular and laboratory measurements by gender**

|  | Nonsmokers  (n= 27) | | Current smokers  (n= 20) | | COPD  (n= 61) | |
| --- | --- | --- | --- | --- | --- | --- |
|  | Men  (n= 12) | Women  (n= 15) | Men  (n= 9) | Women  (n= 11) | Men  (n= 51) | Women  (n= 10) |
| Age, years | 57 ± 7 | 56 ± 9 | 56 ± 10 | 53 ± 6 | 63 ± 7*^§^ | 62 ± 5^¥^ |
| BMI, kg/m^2^ | 29 ± 3 | 25 ± 3* | 28 ± 5 | 26 ± 6 | 28 ± 4 | 25 ± 4⁰ |
| Smoking status |  |  |  |  |  |  |
| Current smokers, n (%) | 0 (0) | 0 (0) | 9 (100)* | 11 (100)^#^ | 12 (23)*^§^ | 4 (40) ^#¥^ |
| Former-smokers, n (%) | 0 (0) | 0 (0) | 0 (0) | 0 (0) | 39 (77) *^§^ | 6 (60) ^#¥^ |
| Smoking history, pack-years | 0 | 0 | 41± 30* | 21 ± 15^#^ | 63 ± 26*^§^ | 68 ± 39^#¥^ |
| Framingham risk score ‡ | 9 ± 6 | 2 ± 2* | 13 ± 4 | 2 ± 2^§^ | 12 ± 6 | 3 ± 2⁰ |
| Spirometric GOLD stage 1/2/3/4  n  (%) | NA | NA | NA | NA | 2/13/16/20  4/26/31/39 | 1/4/2/3  10/40/20/30 |
| FEV_1_, % predicted | 103 ± 11 | 110 ± 12 | 100 ± 9 | 104 ± 12 | 41 ± 18*^§^ | 55 ± 21^#¥‡^ |
| FVC, % predicted | 103 ± 8 | 108 ± 14 | 103 ± 15 | 104 ± 10 | 78 ± 18*^§^ | 88 ± 24^#¥^ |
| FEV_1_/FVC | 77 ± 4 | 79 ± 5 | 75 ± 5 | 79 ± 4 | 40 ± 14*^§^ | 46 ± 11^#¥^ |
| TLC, % predicted | 104 ± 8 | 106 ± 8 | 105 ± 9 | 107 ± 9 | 115 ± 19 | 120 ± 23 |
| RV, % predicted | 104 ± 19 | 111 ± 18 | 100 ± 23 | 118 ± 21 | 185 ± 57*^§^ | 180 ± 68^#¥^ |
| DL_CO_, % predicted | 99 ± 15 | 86 ± 13 | 89 ± 11 | 83 ± 6 | 53 ± 20*^§^ | 50 ± 16^#¥^ |
| PaO_2_, mmHg | NA | NA | NA | NA | 71 ± 11 | 66 ± 9 |
| PaCO_2_, mmHg | NA | NA | NA | NA | 44 ± 8 | 43 ± 6 |
| Systolic blood pressure, mmHg | 127 ± 15 | 121 ± 20 | 131 ± 16 | 116 ±12 | 132 ± 18 | 127 ± 26 |
| Diastolic blood pressure, mmHg | 79 ± 9 | 72 ± 8 | 75 ± 7 | 71 ± 8 | 77 ± 11 | 74 ± 13 |
| Total Cholesterol, mg/dL | 204 ± 20 | 206 ± 31 | 214 ± 39 | 221 ± 39 | 190 ± 32 | 217 ± 34 |
| HDL, mg/dL | 49± 12 | 63 ± 14* | 65 ± 18* | 71 ± 13 | 56 ± 17 | 67 ± 17⁰ |
| LDL, mg/dL | 133 ± 20 | 126 ± 23 | 129 ± 38 | 134 ± 28 | 111 ± 29* | 126 ± 30 |
| Mellitus diabetes, n (%) | 0 (0) | 1 (7) | 0 (0) | 0 (0) | 7 (14) | 0 (0) |
| Lymphomonocytes, x10^5^events | 8.8 ± 1.4 | 8.7 ± 1.4 | 8.9 ± 1.1 | 9.5 ± 3.2 | 8.3 ± 2.3 | 8.9 ± 3.3 |
